# Are There Neurological Symptoms in Type 1 of Gaucher Disease?

**Published:** 2018

**Authors:** Mohammadreza ALAEI, Narjes JAFARI, Farzaneh ROHANI, Farzad AHMADABADI, Rezvan AZADI

**Affiliations:** 1 Department of Pediatric Endocrinology, Faculty of Medicine, Shahid Beheshti University Of Medical Sciences, Tehran, Iran; 2 Pediatric Neurology Research Center, Shahid Beheshti University of Medical Sciences, Tehran, Iran; 3 Pediatric Growth & Development Research Center, Iran University of Medical Sciences, Tehran, Iran; 4 Department of Pediatric Neurology, Ardebil University of Medical Sciences, Ardabil, Iran

**Keywords:** Neurological symptom, Gaucher type 1, Gaucher type 3, Glucocerebroside

## Abstract

**Objective:**

Gaucher disease (GD) is a rare inborn error of metabolism, classified as a lipid storage disorders. This disease is caused by a deficiency in glucocerebrosidase enzyme. It has been classified according to the presence or absence of neurological symptoms into the following types: type 1 non-neuropathic, type 2 acute infantile neuropathic and type 3 or chronic neuropathic. We evaluated neurological symptoms in patients with GD1 and GD3 and compared both of these groups.

**Materials & Methods:**

Eleven patients were identified according to their clinical presentation and the presence of disease confirmed by genetic testing, from 2006-2016, at the Mofid Children Hospital Clinic, Tehran, Iran. We included eight patients with GD 1 and three patients with GD3. Careful neurological examination was performed on these patients during treatment by pediatric neurologist.

**Results:**

Patients with GD1 had some neurological symptoms including cognitive impairment, developmental disability, behavioral disorder, microcephaly and increased deep tendon reflexes (DTR). Of course, neurological signs in patients with type 3 of GD were different and were included seizures, supranuclear gaze palsy, cerebellar signs, and ataxia.

**Conclusion:**

The current nomenclature for 3 types of Gaucher disease does not meet all clinical symptoms. Patients with GD1 display many neurological deficits in young ages not reported adequately earlier.

## Introduction

Gaucher disease (GD) is the most common disease of inborn error of metabolism. It is an autosomal recessive lipid storage disorder, caused by a defect in the gene that encodes β-glucocerebrosidase (*GBA* gene). *GBA* gene is located on chromosome1q21. Almost 300 mutations and polymorphisms in *GBA* have been identified. Most cases are point mutations, insertions, and deletions; splice site alterations and recombination alleles have also been identified (1). Four mutations account for over 90% of disease alleles. The allele mutations N370S, 84GG, L444P, and IVS2+1Gare are common in Ashkenazi Jewish patients (2). Non-Jewish patients exhibit a wider range of genotypes, although two mutations including N370S and L444P are common in both populations (3). Defect in *GBA* induced accumulation of glucocerebroside in the spleen, bone marrow, liver, brain, and lungs (4, 5). 

The onset of disease in type 1 may vary from early childhood to adulthood. Symptoms such as bone pain, bone fractures, splenomegaly, hepatomegaly, and cytopenia (anemia, leukopenia or thrombocytopenia) are common. GD1 is the most common type of this disease. In type 1 of GD, symptoms may not be clinically significant until adulthood. These patients usually present with easy bruises due to thrombocytopenia, fatigue due to anemia, and recurrent infections due to leukopenia. Other abnormalities may also be present, such as abdominal distention or abdominal discomfort due to organomegaly and skeletal disorders such as osteopenia, lytic bone lesions, pathological bone fractures, chronic bone pain, bone crisis, bone infarcts, osteonecrosis and skeletal deformities (1). GD1 has been named non-neuropathic form. However, in past studies, neurologic symptoms have been reported in non-neuropathic GD, too. For example in one study, neurological deficits were mentioned in 30.7% of these patients and included on dementia, psychomotor delay, Parkinsonism, impaired saccadic eye movements and peripheral neuropathy (6).

However, another study reported experiences on neurological manifestations of non-neuropathic Gaucher's disease and concluded recent data may discuss on Gaucher's classification and suggest the existence of a continuum between neuropathic and non- neuropathic forms of the GD disease (7). Another study reported neurologic complications in patients with type 1 of Gaucher's disease, but secondary to systemic features of the illness, such as coagulopathy, or skeletal disease (8). GD2, acute infantile neuropathic form of this disease, manifests as severe neurological deficits that appear around 3 months of age and patients usually die before 2 yr of age not discussed in our study (9).

In GD3, the most common symptoms are skeletal abnormalities, eye movement disorders, respiratory problems, blood disorders and brain involvement. The brain abnormalities manifest gradually, as seizures, cognitive deficits, and poor coordination. Eye movement abnormalities, such as horizontal saccadic eye movements, have also been reported in type 3 (10). 

In this study, we evaluated neurological symptoms in the patients with GD1 and GD3 and compared both of these groups.

## Materials & Methods

Patients with symptoms suggestive of Gaucher disease were screened, selected and followed over a period of 10 yr (since Oct 2006 until Nov 2016) at the Mofid Children Hospital Clinic in Tehran, Iran. The clinical deficits of these patients manifested by blood disorders, skeletal involvement, organomegaly, respiratory problems and/or neurological involvement such as seizures, cognitive deficit, poor coordination, ataxia, developmental disability, nuclear gaze palsy, oculomotor apraxia and so on. In addition to identifying these patients by their clinical manifestations, β-glucocerebrosidase activity on leukocytes and fibroblasts was determined by enzyme assay in wagnester laboratory in German. β-Glucocerebrosidase is an enzyme with glucosylceramidase activity cleaved by hydrolysis, the beta-glucosidic linkage of the chemical glucocerebroside, an intermediate in glycolipid metabolism (11).

After identifying the patients with β-glucocerebrosidase deficiency, genetic testing was performed to confirm diagnosis. A complete physical exam, including a delicate neurological assessment, was performed by Pediatric Neurologist in the Clinic of Mofid Children Hospital. We also recorded the cognitive and developmental status, behavioral disorders and any seizure activity. In physical examination, head circumference, eye movement abnormalities such as strabismus, gaze palsy, oculomotor apraxia, cerebellar function, and DTR were evaluated. Developmental status was assessed by Denver 2 test; cerebellar function was evaluated by clinical tests such as tandem gait, finger to nose, heel to shin, dysdiadochokinesia and so on. 

The data were analyzed by descriptive methods and no statistical testing was applied as observational study.

## Results

Eleven patients with the diagnosis of GD1 (8 patients) and GD3 (3 patients) were included. The enzymatic study showed decreased β-Glucocerebrosidase activity and diagnosis was further confirmed by genetic testing (Table 1).

**Table 1:** The result of B glucocerebrosidase enzyme activity

N	**Type of disorder**	**Genotype**	**Glucocerebrosidase activity**	**Chitotriosidase activity**
1	1	M416r/-	**2**	**3070**
2	1	M416r/-	**1.3**	**3202**
3	1	Homozygote l354v		**4.47**
4	1	R163q/r163q + n188s/n188s	**3.59**	**2947.5**
5	1	Homozygote s400g	**0.7**	**8048.4**
6	1	M416r/-	**1.3**	**2062**
7	1	M416r/-	**1.9**	**11577**
8	1	Homozygote l444p	**1.77**	**3483.9**
9	3	Homozygote l444p	**1.93**	**38131**
10	3	Homozygotel444p/l444p	**2.8**	**6213**
11	3	Homozygotell444p/l444p	**6.17**	**2381**

Eight patients had GD1, and three had GD3. Seven of these patients were female. The mean age of patients was 9.95 yr. The patient’s ages ranged from 5-30 yr old with the mean age at the time of diagnosis being 4.36 yr. All of patients had parents with consanguinity of marriage. Parents were first cousin in ten patients and second cousin in one case. Three patients had a positive family history of similar diseases.

Chief complaints expressed by GD1 patients at the time of presentation consisted as follows: Abdominal distention and discomfort due to hepatosplenomegaly were seen in 75% of patients, epistaxis due to thrombocytopenia in 25% of patients, anorexia and weight loss in 25%, also, limb pain with difficulty walking in one patient. Chief complaints in primary presentation of patients with GD3 were similar to patients with GD1, with hepatosplenomegaly in all of GD3 patients, epistaxis in one patient, and anorexia and weight loss in two patients. Additionally, there was delay in teeth growth in all of them.

Neurological problems were detected in all of eight patients with GD1 and included: cognitive impairment in one patient (13%), developmental delay and microcephaly in four patients (50%), behavioral disorder, mild spasticity and increased DTR in three patients (in 37%). 

On the other hand, neurological findings in three patients with GD3 were developmental delay in all of them as well as cerebellar dysfunction, and disrupted Trendelenburg test in all cases, ataxic gait, the behavioral disorder, myoclonic seizure in one patient. Abnormal eye movements were noticeable in all of the patients with GD3 with convergence esotropia and disrupted saccadic eye movement in one case, esotropia with high pressure in eyes with corrected surgery in one patient, and disrupted saccadic eye movement in horizontal direction and upward gaze in another case.

**Table 2:** Comparing the neurological deficits in patients with type 1 and type 3 of Gaucher disease

N	**Type**	**Age**	**Developmental status**	**Cognitive status**	**Behavioral status**	**Seizure**	**Headcircomfrence**	**Eye movement**	**Cerebellar tests**	**Dtr**
1	1	8	Gross motor delay (walking in 2 years old)	Nl	Nl	Negative	Microcephal		Nl	Nl
2	1	10	Verbal delay (with speech therapy is normalized,now)	Nl	Aggression	Negative	Normal	Nl	Nl	Nl
3	1	5.5	Motor delay (walking in1.5 years old after beginning enzyme treatment)	Nl	Nl	Negative	Microcephal		Nl	Increased
4	1	7	Nl	Nl	Nl	Negative	Nl	Nl	Nl	Nl
5	1	8	Social developmental delay	Cognitive decline	Aggression-restlessness	Negative	Microcephal		Nl	Increased
6	1	8	Nl	Nl	Nl	Negative	Microcephal		Nl	Nl
7	1	30	Nl	Nl	Restlessness-sleep disorder	Negative	Nl	Nl	Nl	Increased
8	1	10	Nl	Nl	Nl	Negative	Nl	Nl	Nl	Nl
9	3	9	Global developmental delay	Nl	Nl	Negative	Microcephal(46.5cm	Strabism-gaze palsy	Abnormal trendelenburg and rumborg test	Increased
10	3	4	Global developmental delay	Nl	Restlessness-sleep disorder	Myoclonic epilepsy	Nl	Strabism-gaze palsy	Ataxia	Increased
11	3	10	Global developmental delay	Nl	Nl	Negative	Nl	Gaze palsy	Abnormal trendelenburg and rumborg test	Increased

## Discussion

We found that all of patients with GD1 presented with abdominal distention and discomfort due to hepatosplenomegaly, epistaxis due to thrombocytopenia, anorexia, weight loss and limb pain. Only one patient manifested with impaired walking. The chief clinical finding in patients with GD3 was similar to patients with GD1, contains hepatosplenomegaly, epistaxis, anorexia and weight loss, as well as a delay in teeth, grow in all of three patients.

Three types of GD have been reported. The most prevalent is type 1 or non-neuropathic with prominent abnormalities such as thrombocytopenia, anemia, organomegaly and skeletal abnormalities, that our patients presented these symptoms, too. Type 2 GD, or acute neuropathic, with a poor prognosis and survival (12). In addition, GD type3, in these type neurological symptoms, commonly manifest late in life as compared to type 2, and include abnormal eye movements, ataxia, seizures, and dementia, and patients live until their third or fourth decade of life (13-15). Our patients with GD3 presented by hepatosplenomegaly, thrombocytopenia, anemia, skeletal muscle dysfunction plus central neurological involvement.

Neurological problems in our patients with GD3 included developmental delay, behavioral disorder, myoclonic seizure, and cerebellar dysfunction; disrupted Trendelenburg test and abnormal eye movements. Ataxic gait in one patient, convergence esotropia and disrupted saccadic eye movements in another case, high pressure in the eye with history of twice eye surgery for strabismus correction in one patient, as well as disrupted saccadic movements in the horizontal and upward direction identified in another case.

Dysfunction of horizontal saccadic eye movement, brainstem bleeding or glioma has been reported commonly in type 3 of more gauche disease. Impairment of horizontal and vertical saccadic eye movement is seen in later stages of GD type 3 or Niemman pick type C (7). GD3 can be presented by complex cerebellar symptoms such as cerebellar ataxia, intention tremor and Progressive myoclonic epilepsies (PMEs) (16-18). 

In our study, patients with GD1 presented with a variety of neurological problems such as cognitive impairment in one case, developmental delay and microcephaly in 4 patients (50%), behavioral disorder, mild spasticity and increased DTR in 3 patients in mean age of 9.95 yr. Cognitive impairment, Parkinsonism, tremor and gait impairment in GD 1 are reported (19). Patients with GD type 1 might develop some late-onset neurological deficits (4). Neurological problems have been reported in 30.7% of patients with GD1 in adulthood, and included on: dementia, psychomotor delay, Parkinsonism (6).

Most of previous studies have been done in adult patients, so they often noticed early dementia and Parkinsonism in neurological presentation of their GD1 patients, but we studied on children and mean age of our patients at the bingeing of assessment was 4.36 yr, so our patients presented with different pattern with spasticity, developmental delay, microcephaly, and so on.

Another study reported neurologic complications in patients with GD1 secondary to systemic features of the illness, such as CNS bleeding due to coagulopathy, or abnormal gait induced by skeletal disease (8). However, our patients had spasticity, increased DTR, microcephaly and other signs that were not justified by primary problems such as coagulopathy, or skeletal disease.

Another study reported neurological manifestations of GD1 and according to recent information deducted that may discuss Gaucher's classification and the existence of a continuum between neuropathic and non- neuropathic forms of the GD disease (7). Thus, according to our assessment, patients with GD1 have variety of neurological problems such as developmental delay, microcephaly, behavioral disorders, spasticity, and so on, although some kinds of neurological involvements such as myoclonic seizure, supranuclear gaze palsy, cerebellar sign, and ataxia, are more typically seen in GD3 patients.

However, unlike previous studies that mentioned GD type 3 and type 1 differentiated by existence of neurological involvements in type 3, we found that type 1 could also be presented with some neurological deficits. Therefore, the neurological symptoms are not the important criteria for differentiation between different types of more gauche disease, and genetic testing is required for confirmation of diagnosis and determining a treatment plan.

**In** **Conclusion, **current nomenclature for 3 types of Gaucher disease does not meet the clinical symptoms presented in all the patients. Patients with GD1 display many neurological deficits in young ages which less reported in prior studies. We certainly need further studies in larger populations to identify clearly more patients with the same deficits as what our study indicated. 
